# An intervention to promote positive homeworker health and wellbeing through effective home-working practices: a feasibility and acceptability study

**DOI:** 10.1186/s12889-023-15347-x

**Published:** 2023-03-31

**Authors:** Samuel Keightley, Myanna Duncan, Benjamin Gardner

**Affiliations:** 1 Department of Psychology, Institute of Psychiatry, Psychology and Neuroscience, King’s College London, De Crespigny Park, London, SE5 8AF UK; 2 Department of Psychology, University of Surrey, Guildford, GU2 7XH Surrey UK

**Keywords:** Homeworking, Health-behaviour, Wellbeing, Digital intervention, Feasibility, Acceptability

## Abstract

**Background:**

In the wake of Covid-19, the prevalence of working from home (‘home-working’) is expected to rise. Yet, working from home can have negative health and wellbeing impacts. Interventions are needed to promote effective ways of working that also protect workers’ health and wellbeing. This study explored the feasibility and acceptability of an intervention intended to promote home-working practices that would protect and promote health behaviour and wellbeing.

**Methods:**

An uncontrolled, single-arm mixed-methods trial design was employed. Forty-two normally-office-based UK workers, working from home between January–February 2021 (during the Covid pandemic), consented to receive the intervention. The intervention: a digital document offering evidence-based recommendations for home-working in ways conducive to health behaviour and wellbeing. Feasibility and acceptability were quantitatively indexed by: expressions of interest within 1 week (target threshold ≥ 35); attrition across the one-week study period (threshold ≤ 20%); and the absence of any apparent detriments in self-reported physical activity, sedentary behaviour, snacking, and work-related wellbeing prior to and one week after receiving the intervention. Qualitative think-aloud data, obtained while participants read through the intervention, and analysed using reflexive thematic analysis, explored acceptability. Semi-structured interviews conducted one week after intervention exposure were content-analysed to identify whether and which behaviour changes were adopted.

**Results:**

Two feasibility criteria were met: 85 expressions of interest indicated satisfactory intervention demand, and no detriments were observed in health behaviours or wellbeing. Forty-two participants (i.e., maximum capacity for the study; 26 females, 16 males, aged 22–63) consented to take part. 31% dropped out over the one-week study period leaving a final sample of 29 (18 females, 11 males, aged 22–63), exceeding identified attrition thresholds. Think-aloud data showed that participants concurred with intervention guidance, but felt it lacked novelty and practicality. Follow-up interviews produced 18 (62%) participant reports of intervention adherence, where nine recommendations reportedly prompted behaviour change in at least one participant.

**Conclusions:**

Mixed evidence was found for intervention feasibility and acceptability. Whilst the information was deemed relevant and of value, further development is required to increase its novelty. It may also be more fruitful to provide this information via employers, to encourage and emphasise employer endorsement.

**Supplementary Information:**

The online version contains supplementary material available at 10.1186/s12889-023-15347-x.

## Background

Before 2020, organisations’ increasing reliance on digital technology had prompted a steady rise in employees ‘working from home’ (i.e., “home-working”; [1, 2]. The Covid pandemic, and associated lockdowns that led many normally office-based employees to work from home, further increased the prevalence of home-working [3]. The pandemic also led many organisations to recognise that employees can work as productively at home as in the office [4]. Home-working is forecast to become common through ‘hybrid’ working arrangements that combine on-site and home-based working patterns [5]. However, home-working may have consequences for individual’s health-related behaviours and wellbeing [6–10]. Efforts to promote health and wellbeing among employees tend to focus on and be administered in the workplace [11]. Few interventions exist that explicitly promote health behaviour and wellbeing when working from home [12].

The potential impact of working from home, rather than the office, on health behaviour and work-related wellbeing can be understood by adopting a goal-based perspective on work practices [13, 14]. For organisations and their employees, the priority during working time is typically to be productive [15]. Pursuing the goal of productivity often incurs behaviours that incidentally improve or diminish employee health and work-related wellbeing [16]. For example, many office-based employees are required to be on-site to complete their work tasks, which in turn requires them to commute to and from the workplace. For those that use non-car travel modes (e.g. cycling, walking), the act of travelling to and from work often incidentally incurs physical activity. For example, a study of London office workers showed that, on workdays, step count was highest during the morning and evening commute [17]. Commuting can also support individuals’ work-life balance because it offers an opportunity to psychologically demarcate and transition between work and home settings [18]. Similarly, many office-based work tasks—such as collecting printing from a communal printer or accessing refreshments – require physical movement around the workplace [16, 19]. The physical layout of the workplace can encourage or inhibit physical activity: for example, teams that work across multiple floors, have accessible staircases, height adjustable workstations, and standing permissive meeting rooms, tend to be more physically active [20]. The social environment within the workplace can also shape health-related behaviours. For example, in the workplace, people are more proximally exposed to localised office cultural norms that promote and encourage shared healthy workday eating behaviours [21]. The physical presence of colleagues in the office space also encourages movement to other desk spaces or meeting rooms during the workday, to facilitate face-to-face interactions [22].

When individuals work from home, the instrumentality of many of their workplace-oriented behaviours for achieving work goals changes, which in turn affects engagement in health-related behaviours and work-related wellbeing. For example, commuting becomes unnecessary, which can reduce daily physical activity and limit individuals’ ability to psychologically transition between work and leisure time. Additionally, the spatial environment of the home is likely smaller compared to a typical office, so attending to working tasks and communicating with colleagues requires less physical activity. Accordingly, research shows that those working from home instead of the workplace experience a decrease in daily physical activity [10, 23, 24], increased sedentary behaviour [25], and greater difficulties achieving work-life balance [26]. A recent qualitative study of homeworkers during the stay-home Covid-19 lockdowns showed how adaptations to work-related practices affected health behaviours and wellbeing [9]. Participants reported sitting more and moving less due to the diminished need to move away from one’s computer when attending to daily work tasks. They also reported a physical and psychological blurring of work and home life boundaries, stemming from the continued presence of work-based visual cues (e.g., work computers) during leisure time, and a tendency to work for longer hours. Perhaps consequently, they reported difficulties ‘switching off’, and reductions in sleep quality. Dietary behaviour was also disrupted by the home environment, with greater proximity and accessibility of food, and situation-specific habitual home-based behaviours reportedly causing increases in snacking.

Interventions are needed to support health behaviour and work-related wellbeing when working from home. Yet, at the time that we designed the present study (July 2020), to our knowledge no evidence-based interventions or initiatives existed to promote health behaviours and wellbeing for normally office-based workers when working from home. Drawing on Keightley et al. (2022), we developed an intervention (during the Covid pandemic of 2020) to promote work practices that facilitate (or at least do not hinder) achievement of work-related goals, while also incidentally shielding or improving health behaviour and work-related wellbeing. Our intervention aimed to emphasise the importance of health and wellbeing beneficial homeworking practices. Our aim was to design an intervention for delivery by employers as an onboarding tool for new staff, and as annual refresher training for existing staff. The present study, data for which were collected during the Covid pandemic (Jan-Feb 2021), was designed to assess and explore the feasibility and acceptability of prototype content for this intervention among normally office-based workers who were working from home due to the pandemic.

Intervention feasibility captures whether an intervention can be delivered as intended [27], and acceptability broadly encapsulates whether potential recipients are willing and able to receive and adhere to the intervention (See [28]. Feasibility and acceptability are precursors of intervention effectiveness; an intervention that cannot feasibly be implemented, or is unacceptable to the target audience, is unlikely to be effective. Feasibility and acceptability assessments are important for informing decisions about whether to progress to subsequent phases of the intervention development process [29]. The present study, which employed a mixed methods design, was designed to inform a decision about whether to progress to a more rigorous evaluation trial.

## Method

### Participants & procedure

An uncontrolled, single-arm mixed-methods trial design was employed. The project was run as a postgraduate research project during the Covid pandemic (2020–21), the time and financial constraints of which prompted us to trial intervention content in a different context (i.e., delivered individually to workers) to that in which we envisaged the full intervention being implemented (i.e., delivered by employers). Participants were recruited during January and February 2021 via a study advert published on social media (Twitter, LinkedIn), and in an internal circular email to staff at an inner-city university in the south of England. A £20 Amazon voucher was offered as an incentive for completing the 1-week study. Participants were eligible to take part if they were: aged 18 or above; full time employed; working from home at the time of the study; with no caring responsibilities for pre-teen children or older adults. The study advert contained a survey link to a questionnaire where participants self-declared eligibility and completed measures capturing demographic information (age, gender & average hours worked^1^) and self-reported health-related behaviour and work-related wellbeing. With 35 participants deemed an adequate sample size for assessing feasibility and acceptability among study completers [30], a total of 42 participants were consented in anticipation of 20% attrition.

Next, participants arranged a time to take part in an online ‘think aloud’ interview. The interview involved participants talking aloud as they read over the intervention document for the first time, followed by some brief semi-structured questions to probe their responses. Participants had full and continual access to the intervention document after the ‘think aloud’ interview. One week after the ‘think aloud’ interview, participants were sent an email that included a link to a set of follow-up health behaviour and wellbeing measures, and an invitation to attend a semi-structured follow-up interview

### Intervention

The intervention was developed between August-October 2020, based on theory and evidence around how adaptations to work practices, made in response to having to work from home during the early days of the Covid-19 pandemic, had shaped office workers’ health behaviour and wellbeing ([9],See too [31]). For example, in an interview study conducted in April–May 2020, UK office workers reported that the removal of the daily commute had reduced their physical activity, and prevented them from psychologically transitioning from work to leisure time, which made it difficult to ‘switch off’ from work demands [9]. Similarly, a shift to digital forms of work-related communication, and a greater perceived frequency of online meetings, reportedly prolonged increased time and limited opportunities for breaks [9].

We developed our intervention in line with the COM-B model [32, 33], which proposes three fundamental determinants of behaviour: capability, opportunity, motivation. Guided by emerging research on experiences during the pandemic [9], we conducted an informal ‘behavioural diagnosis’ to identify which of the three determinants posed significant barriers to health-conducive work practices, and so should be targeted by our intervention [33]. We assumed that during the pandemic, normally-office-based workers retained sufficient physical and psychological capacity (i.e., *capability)* to engage in work practices conducive to health behaviours and wellbeing, but failed to identify or capitalise on the *opportunities* available to enact such behaviours in the home environment. We also identified *motivation* as problematic, because previous research suggests that workers viewed the goal of protecting health behaviour and wellbeing as conflicting with prioritised productivity goals when working from home (e.g. [9, 14, 34]. Our intervention therefore sought to *motivate* home-workers by highlighting example specific home-working practices that support health behaviour and wellbeing while also facilitating (or at least not hindering) productivity, and to identify and seize *opportunities* to engage in such behaviours in the home-work environment.

Recommendations included in the intervention were drawn from previous evidence-based guidance for home-working practice [31], and strategies experienced by home-workers as useful for promoting health behaviour and protecting wellbeing [9]. Example *motivational* strategies included: creating a dedicated workspace to psychologically separate home and work tasks; bookending working hours with physical activity (i.e., a ‘mock commute’) to allow psychological transitioning between home and work activities; planning working hours and adopting strict timekeeping to ensure consistent start and stop times, so minimising overworking and protecting work-life balance; and making distractions and snacks less physically accessible, to maintain focus and minimise unhealthy snacking. Example strategies promoting *opportunities* included: taking movement breaks (e.g., brief walks) to allow contemplation of difficult tasks while reducing sitting and promote physical activity; taking walks when attending online meetings, to encourage physical activity; and identifying work tasks that can be undertaken while standing, to reduce sitting time.

An interactive PDF delivery format was chosen because this is a widely used, familiar information delivery format among office workers, often used during staff onboarding, orientation, or refresher training [35]. We anticipated that the PDF format would therefore be more conducive to embedding into existing organisational practice. Throughout the intervention document, participants were able to click on icons, giving them access to information which sequentially extended from the core intervention content. Icon information included relevant research examples, quotations, and behaviour change tips.

Additional file 1 provides a comprehensive overview of intervention content as mapped to behaviour change techniques from the Behaviour Change Technique Taxonomy v1 [36], with example screenshots presented in Additional file 2.

### Data collection

#### Quantitative data

All measures were self-reported. Unless stated otherwise, all behaviour and wellbeing measures referred to the previous 7 days.

##### Health behaviour

**Physical activity** was measured using the moderate physical activity (MPA) and walking items from the IPAQ short form, which has been shown to be reliable across many previous studies [37]. The standardised physical activity definitions of the IPAQ short form were presented for comprehension. These items prompted participants to identify on how many days in a working week they engaged in MPA or walking (e.g., “On how many workdays did you do moderate physical activities?”). For any answers above zero, participants were prompted to report the average hours/minutes spent in these activities per day (e.g., “How much time did you usually spend doing moderate physical activities on one of those workdays?”).

**Sedentary behaviour** was measured utilising one item from the IPAQ short form, relating to time spent sitting during workdays (“How much time did you spend sitting on a workday?”).

**Sleep quality** was assessed using two items adapted from the Leeds Sleep Evaluation Questionnaire (LSEQ; [38], a reliable measure that has been validated against objective sleep markers [39]. One item measured ‘getting to sleep’ (GTS; “Over the last 7 days, how would you describe the way you currently fall asleep in comparison to usual?”; “More difficult than usual” [0] to “Easier than usual” [10]) and one assessed ‘overall sleep quality’ (SQ; “Over the last 7 days, how would you describe the quality of your sleep in comparison to usual?”; “More restless than usual” [0] to “Calmer than usual” [10]).

**Snacking frequency** was assessed through a single item, adapted from Pavey and Churchill [40]: “How often did you eat high calorie snacks on an average workday?” (‘Not at all’ [1] to ‘Very often’ [5]).

##### Wellbeing

**Work-related wellbeing** was assessed using the second short form version of The Copenhagen Psychosocial Questionnaire (COPSOQ II short form;,[41], which has been shown to reliably assess four domains of wellbeing [42]. ‘Work life conflict’ was assessed using a single item (“Do you feel that your work drains so much of your energy that it has a negative effect on your private life?”; ‘Yes, certainly’ [1] to ‘No, not at all’ [4]). ‘Burnout’ was assessed by two items (e.g., “How often have you felt worn out?” [Burnout I] & “How often have you been emotionally exhausted?” [Burnout II]; ‘All the time’ [1] to ‘Not at all’ [5]). ‘Stress’ was assessed via a single item (e.g., “How often have you been stressed?”; ‘All the time’ [1] to ‘Not at all’ [5]). ‘Job satisfaction’ was assessed by a single item (“How pleased are you with your job as a whole, everything taken into consideration?”; ‘Very satisfied’ [1] to Very unsatisfied’ [4]).

#### Qualitative data

##### Think aloud interview

Each ‘think aloud’ interview was conducted by one of three undergraduate research students (two female, one male) trained by a senior qualitative researcher (author BG). To assure the data collection protocol was followed correctly, the first two interviews run by each student were observed by SK, a male doctoral research student. Participants met online (via Microsoft Teams, Skype, or Zoom) with researchers where they were briefed on the ‘think aloud’ concept before seeing the intervention. During the online meeting, participants were sent the intervention document and asked to share their screens. Participants were explicitly instructed to read through the information out loud in chronological order whilst offering their thoughts and impressions of the information presented to them (See ‘Additional file 3’). Following the ‘think aloud’ task, participants were asked questions designed to further probe their impressions of the intervention, specifically relating to informational content, format, comprehension, and perceived areas in need of improvement.

##### Follow up interview

In the one-week follow-up interview, which was conducted online or by phone, participants were asked whether and how they made changes to their working practices based on the information presented to them during the think-aloud interview. Topics included experiences of implementation, adherence, how the intervention document was used, and views on the intended use of the digital document in organisational settings (see ‘Additional file 4’ for follow up interview schedule). All follow up interviews were conducted by SK.

### Feasibility and acceptability assessment and analyses

#### Quantitative data

Feasibility was assessed via criteria relating to expressions of interest in participating in the intervention study, and sequential attrition during the one-week study period. Acceptability was assessed via observed changes between baseline and one week follow-up for the behavioural and wellbeing measures.

Established sampling and attrition thresholds currently do not exist within the acceptability literature [43]. In accordance with suggested sample sizes for pilot studies assessing feasibility [30, 44], we set a threshold of at least 35 *expressions of interest* received within a week of publishing the study advert to indicate adequate study feasibility [45].

**Fig. 1:**
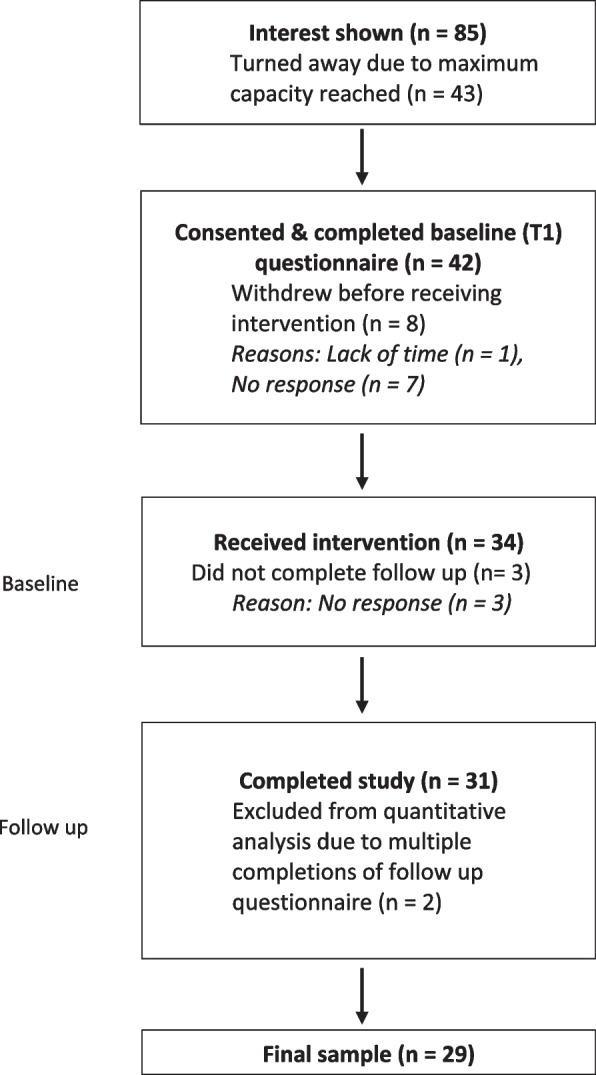
Participation flow diagram

*Attrition* was described via percentage dropouts calculated at each stage of the study procedure (see Fig. 1). An overall attrition of ≥ 20% signalled feasibility issues.

*Potential for harm* was assessed via inspection of behaviour and wellbeing changes between baseline and follow-up. Specifically, we visually inspected behavioural and work-related wellbeing measures and treated the intervention as acceptable if there was no apparent decline in any behaviour or wellbeing measure between baseline and follow-up. Given the small sample size and likely minimal statistical power, our analysis of pre-post changes solely aimed to describe potential trends, rather than establish statistical significance. Nonetheless, for comprehensiveness, pre and post mean comparisons were conducted using inferential statistics, and *p*-values are reported.

#### Qualitative data

Digital audio of think-aloud and follow-up interviews was automatically transcribed using Otter.ai [46]. Transcripts were manually error checked by SK. All qualitative analyses were organised using Nvivo 12 [47].

For the think-aloud interviews, acceptability was explored using ‘codebook’ thematic analysis [48]. Analysis followed a five-stage process of familiarisation, coding, theme extraction, theme review and theme naming [49, 50]. The ‘codebook’ method involved authors SK and BG independently coding three transcripts and through comparative discussion, establishing an initial thematic framework, which was subsequently applied and refined by SK to the remaining transcripts [51]. Through regular supervision and discussion, the thematic framework was iteratively developed whilst SK analysed the remaining transcripts. ‘Codebook’ methods were adopted because they were deemed most pragmatic, given time constraints on the project, as they allowed for initial insights from analysis to be drawn on throughout subsequent coding [51].

We intended to analyse follow up interviews using thematic analysis procedures to understand details of participant behavioural adherence. However, during familiarisation, it transpired that the interview data lacked the ‘thick description’ required for an in-depth analysis [52]. In response, a method of summative content analysis was employed to draw indications of guidance adherence from the follow up interview data [53]. Reports of engaging in behaviours recommended by the intervention were identified, counted, and descriptively summarised using frequencies and percentages. Presented data identified particular behaviour change recommendations that demonstrated acceptability through reported adherence during the one-week follow up period.

## Results

### Quantitative

#### Expression of interest

Within one week of recruitment going live, a total of 85 individuals expressed their interest to take part in the advertised intervention study. This surpassed our target of 35 expressions of interest, so met our feasibility criterion.

#### Sample description and attrition

As seen in Fig. 1, 42 participants consented to take part and completed baseline (T1) measures (26 females [62%], 16 males [38%]; age range 22 – 63 years, M = 33, SD = 9). Of the 42, seven (17%) failed to respond when arranging the ‘think aloud’ protocol, and one (2%) stated they did not have the time to take part. Thirty-four individuals (21 females [62%], 13 males [38%], age range 22 – 63, M = 35, SD = 9) successfully arranged and attended the ‘think aloud’ protocol session. Of the 34, three (9%) failed to respond when arranging the follow up interview session, and two (6%) completed the follow up measures multiple times and inconsistently, so were removed from analysis. A total of 29 participants (18 females, 11 males; age range 22 – 63, M = 34, SD = 9) belonging to a range of industries (e.g. finance, communications, higher education, public health, recruitment, and charity) successfully attended and completed all stages of the acceptability study.

Attrition between baseline and follow-up was 31%, which exceeded the 20% threshold. This feasibility criterion was not met.

#### Behavioural & work-related wellbeing changes

As Table 1 indicates, there was no visible detriment in any of the observed variables. Whilst none of the mean scores significantly differed between baseline and follow up (minimum p = 0.24), data indicated that the intervention had no potential for harm, therefore the acceptability criterion was met. Notably, an apparent tendency towards improvement was observed in per week MPA and walking.

**Table 1:** Self-report physical activity, sedentary behaviour, sleep, diet, and work-related wellbeing mean and median scores (*n* = 29) from pre (T1) to post (T2), paired samples t-test t scores, *p*-values

	T1 Mean (SD)	T2 Mean (SD)	T1 Median (Range)	T2 Median (Range)	t (*p*-value)
**Physical Activity**					
MPA (IPAQ) days / week	2.41 (2.29)	2.52(2.08)	3 [7]	2 [7]	0.32 (0.751)
MPA (IPAQ) mins / week	28.28 (24.06)	37.59 (28.9)	30 [60]	40 (120)	1.597 (0.121)
Walking (IPAQ) days / week	3.76 (2.43)	4.03 (2.32)	4 [7]	4 (7)	0.812 (0.424)
Walking (IPAQ) mins / week	56.31 (43.24)	65.34 (48.37)	61 (150)	60 (180)	1.193 (0.243)
Sitting (IPAQ) mins / week	1017.69 (1117.54)	1033.97 (1358.14)	630 (4440)	600 (5700)	0.063 (0.95)
**Sleep**					
LSEQ Ease of falling asleep	4.41 (1.722)	5 (1.69)	5 (7)	5 (8)	1.3 (0.204)
LSEQ Sleep quality	4.38 (1.72)	4.90 (1.68)	5 (8)	5 (7)	1.168 (0.253)
Snacking frequency					
Average snacking per day	3 (1.1)	3.07 (0.96)	3 (4)	3 (4)	0.402 (0.691)
**Work-related Wellbeing**					
COPSOQ—WFC	2.45 (0.99)	2.66 (0.936)	2 (3)	3 (3)	1.440 (0.161)
COPSOQ—Burnout I	3.03 (1.27)	3.31 (1.17)	3 (4)	4 (4)	1.612 (0.118)
COPSOQ—Burnout II	3.28 (1.22)	3.38 (1.21)	3 (4)	4 (4)	0.550 (0.586)
COPSOQ—Stress	3.28 (1.16)	3.17 (1.14)	3 (4)	3 (4)	0.571 (0.573)
COPSOQ—Job satisfaction	2.41 (0.95)	2.38 (0.82)	2 (3)	2 (3)	0.273 (0.787)

*MPA* Moderate physical activity, *IPAQ* International physical activity questionnaires, *LSEQ* Leeds sleep evaluation questionnaire, *COPSOQ* Copenhagen psychosocial questionnaire

### Qualitative

#### Think aloud

Three acceptability-related themes were identified: ‘Acceptance and elaboration’, ‘Resistance to content’, and ‘Effectiveness of intervention communication’.

##### Theme 1: Acceptance and elaboration on content

Several participants appeared to agree with the information and behavioural guidance, indicating that it was personally relevant:

“Yeah, I agree with that. Commuting means less physical activity. I normally walk to work. So I like to start my day with a walk. Go out and get some fresh air.”; *P31.*

Think-aloud reactions to the intervention information often produced anecdotal confirmations of the home-working behavioural health risks presented:

*“Think that's really, really true. Especially for me being sat at home all day, just working. The only sort of activity I do around the house is going to the kitchen to make lunch or to make a coffee. I think we really do miss out on extra physical *activities* like commuting and moving around the office”; P7.*

Some participants found the content and the behavioural guidance novel, offering them new insights related to home-working:

*“[Creating your workspace your work zone and trying to break your workday] […], *going* for a walk before you start work or doing something you know, to mark the end of your working day. So those things I haven't really thought about before”; P18.*

Many participants expressed a willingness to implement suggested behaviours into their own working patterns:

*“Ah I never even think about. For online meetings. I’m always sat in exactly the same position. Same with taking phone calls. I could easily be moving about when I’m talking.”; P25.*

Participants also described personal experiences that built upon the guidance provided, offering up similar alternatives to suggested behaviours:

*“[I] totally agree with [the tip about creating your own personal space by plugging in headphones and listening to music]. I use another weird thing […] *because* I don't like listening to music whilst while I'm working. Because I get distracted by that and focus too much on music. So I just put earplugs in.”; P14.*

Participants reported that they were already engaging in many of the behaviours recommended by the intervention. They also referred to specific contexts in which they had already experienced benefits that the guidance aimed to offer:

*“100% agree […] Putting [time to focus on specific tasks] in your calendar, it helps. But it's not just that, what does help is when it's in your calendar on outlook is the little box that comes up to remind you in 15 min. And it's kind of like “Wow, I've been working on this so long”, and that this meant that “Okay, I get a break in a minute, I have to move on to this other task in 15 min”. So I agree with that”; P30.*

For some participants, the guidance was deemed helpful because it reinforced the need for actions that they were already taking:

*“[Putting your work stuff away at the end of the day, creating time that you can kind of fake commute and walk in the morning in the evening], just to kind of shut down for the day or start your day. I've been doing that recently in the past few weeks and I can really relate to that and found it a benefit to myself. […] That was a really good example of something that I've really benefited from.”; P13.*

##### Theme 2: Resistance to intervention

Several responses indicated a lack of acceptability of the information provided as well as for some of the suggested behaviour change methods. For example, some doubted the plausibility of intervention content relating to greater day time standing and movement:

*I can't do any work tasks while standing. And my headphones, so when I take calls, *some* of my calls are through Teams. So I have a headset which literally ties me to my computer. So it’s not likely that I can walk around and quite often I need to see what is on the screen.; P32.*

Participants thought that some of the guidance lacked applicability to personal and specific circumstances or situations. For example, some participants highlighted that the limited access to physical space or desk based equipment in their homes meant that the guided information was not applicable to them:

“*It’s difficult to create a work zone when you only have one living room and one *bedroom*.”; P29.*

*[Keep your lower back properly supported. Adjust your seat, you should be able to use the keyboard with wrists and forearms straight and level with the floor.] “That's impossible because my table’s too small and I don't have a proper chair but okay.”; P19.*

Participants also sometimes rejected tips that were deemed to lack credibility in adjusting unhealthy behaviours, such as taping cupboard doors shut to reduce daily snacking:

*“I wasn't keen on [the recommendation to lock tempting foods away]. I don't know if it's practical. I couldn't put a lock on any of my cupboards […], and taping *them* just seems a bit, I don't know, like, a bit harsh”; P12.*

Some participants felt that the guidance was not practical to follow alongside their daily working practices and perceived recommendations as a burden to their productivity:

*“if I [stood up], I wouldn't get two hours more work done. And then I'd be under more pressure and stress from my, the stakeholders I report into because they wouldn't be happy that things weren't getting done. And it's that fine balance *between* looking after yourself and being in a position where you feel like you can do that.”; P21.*

Participants’ comments generally suggested that they felt the guidance lacked novelty for them (*“I don't think I learned anything new”; P2)*, as they already knew much of it. Many participants felt that the guidance was being provided ‘too late’, given their months of experience of working from home during the pandemic:

*“I'm not sure if there's many people who haven't already thought of this. I guess maybe at the beginning of the pandemic, when we were all sent home, this is the kind of stuff that people might have found useful”; P9.*

##### Theme 3: Effectiveness of communication

Responses indicated various ways in which the information could have been more clearly presented and so communicated.

Most participants expressed a favourable response to the intervention design and content presentation. Specifically, participants positively described the layout, length, format, and functionality of the interactive PDF document (*“It was good. It was really easy to navigate. And the top tips were useful because it gave a bit more information, but in quite a clear and accessible way”; P10*), whilst others favoured the document’s length with regards to their realistic and expected ability to engage with the document:

*“It *doesn't* contain too much of useless information, like some of them do,[…] and it's written in a clear and easy language, which is another positive aspect, because our workload and mental workload is already kind of exhausted.”; P14.*

The inclusion of a page count on the intervention document was, however, discouraging for some, as it suggested that the document was prohibitively long:

*“I'm on page 18 and it says ‘out of 42’. And I thought, “Oh no it’s going to take forever”. So that was a bit of a distraction. A little bit, but then I realized it wasn't 42 slides.”; P30.*^2^

Some participant responses indicated difficulties in accessing the information as intended. Specifically, the digital intervention document was designed with interactive pop out functionality (when clicked on), allowing participants to view further information (e.g. behavioural tips, quotes, and study references). Participants however commonly sped through the document without clicking on these pop outs, leaving a significant portion of the guidance unseen:

*“I just felt like I was forgetting to click on some of the top tips and things. So I don't know. *Yeah*, that was probably I may have missed some information, just because I forgot to click on those.”; P22.*

#### Follow up interview—Summative Content Analysis

As seen in Table 2, of the 29 participants who completed the follow-up interview, 18 (62%) described having made changes to their behaviour in response to one or more of nine specific intervention recommendations. The three most popular behaviour change recommendations with reported adherence indicated participant’s acceptance with attempts to increase daily physical movement, initiate more breaks when working, and a proclivity towards ways of drinking more water during the day. Whilst posture appeared to be addressed by some, the remaining five intervention recommendations were only reported to be attempted by one or two participants.

**Table 2:** Specific recommendations that reportedly led to behaviour change, among those reporting changing their behaviour (*N* = 18; 62% of sample)

**Specific recommendation**	**Quotation example**	**N reporting following recommendation**	**%** **Of participants reporting** **changed behaviour (***N* **= 18)**	**%** **Of all participants (***N*** = 29)**
*Make moving a habit*	*“it's definitely made me more cognizant of the amount of time spent sitting for out the day yet made an active effort to just get up and break up those prolonged periods. of sitting.”*	5	28%	17%
*Take breaks*	*“I've been taking regular breaks throughout the day.”*	5	28%	17%
*Stay hydrated*	*“one thing I have been one thing I have been really trying hard to do is, the tip about getting up and getting a small glass of water. So then. So then you have to keep getting up to refill it.”*	4	22%	14%
*Posture*	*“One of the main things I've definitely taken away from it was posture. And I'm quite for that I kind of realised quite quickly that I'm slouched over, my legs are crossed. So over the past week, I've just been a little more mindful”*	3	17%	10%
*Going into sleep mode*	*“I think the one other thing I was able to do was to try and limit phone use like half an hour before bed”*	2	11%	7%
*Creating work boundaries*	*“Well, I really tried, actually, to switch off my computer at half five, you know, eight hours after I started work.”*	2	11%	7%
*Add it to the calendar*	*“T*he calendar aspect of booking into certain things to do at certain points of day, so I can kind of stick to a routine […] *it's kind of reinvigorated me to stick to my calendar”*	1	6%	3%
*Create a work only space*	*“We had a bedroom that became free, because our daughter went back to uni. So and because of that, and also the conversation, I've moved the, the kind of work and office into the bedroom.”*	1	6%	3%
*Marked start and end of day*	*“I will remember that it said that by and I, like I was going for morning walks before, and I think I've stuck to them a little bit better.”*	1	6%	3%

## Discussion

This mixed method study explored the feasibility and acceptability of an intervention designed to promote ways of working effectively at home while protecting health behaviour and wellbeing. The intervention met some, but not all, feasibility and acceptability criteria. Specifically, there were sufficient expressions of interest in participating in the study, and no detriments were observed in quantitative health behaviour and wellbeing indicators, and there was some tentative indication of a tendency towards greater MVPA and walking one week after receiving the intervention. Overall attrition, however, exceeded our target threshold of 20%. Furthermore, qualitative data suggested that, while participants concurred with the gist of the information presented, and some adopted some of the recommended behaviours, many had already spontaneously adopted such behaviours during the Covid-19 pandemic, so the guidance lacked novelty. Overall, acceptability was mixed. Further development work may be required to enhance the novelty and appeal of the intervention prior to undertaking more rigorous evaluation work.

We assessed acceptability and feasibility quantitatively against three criteria—intervention demand, attrition, and the absence of detriments in health behaviour and wellbeing outcomes – and used qualitative data to explore experiences of and reflections on the intervention. Intervention demand was high; we received far more expressions of interest in response to the study advert than we aimed for, exceeding our target threshold of 35. Additionally, health behaviour and wellbeing did not decline following the intervention. However, 31% of participants dropped out of the study over the one-week study period. While there are no standardised thresholds for assessing acceptability [43], and evidence suggests attrition rates of around 50% are standard for web-based interventions [54, 55], our findings indicate that around a third of those exposed to our intervention study disengaged. It should be noted however that most participants dropped out after consenting but prior to receiving the intervention. Only three (9%) participants dropped out after receiving the intervention, indicating a potentially acceptable level of intervention engagement.

Our think aloud data, obtained as participants read through the intervention information for the first time, offered potential reasons for lack of engagement. Although participants indicated agreement and demonstrated a clear comprehension of the intervention material, some of the recommendations appeared to lack novelty. We drew on insights from research conducted in the Spring 2020 pandemic regarding how office workers had adapted to home-working, and so expected our intervention to be timely, novel and informative. Yet, participants appeared to have accrued homeworking experience during the Covid-19 pandemic to a degree that the knowledge and guidance offered by the intervention was all too familiar and perceived to have been delivered too late. A lack of informational novelty has been shown to reduce engagement with behaviour change suggestions in interventions [56].

Additionally, many indicated that they would find it difficult to engage in some of the guided advice, because it failed to recognise practical barriers to adherence. For example, recommendations to use physical space for work-life demarcation were deemed unfeasible by those with smaller homes. Evidence suggests that those who do not have dedicated workspaces at home are at a higher risk of negative health and wellbeing behavioural outcomes [10]. This underlines the necessity for home-working advice to acknowledge space constraints and to offer clearer applied examples of implementation which better emphasise the benefits of the space management strategies presented. It may be helpful and more acceptable for guidance to be tailored to users’ circumstances.

Some participants felt that adopting some of the intervention recommendations would be burdensome and would inhibit their productivity. For example, the suggestion that workers should stand for a total of two hours per day was dismissed as impractical by some. This suggests that some recommendations failed to achieve our aim of promoting health behaviours and wellbeing in a way congruent with the pursuit of daily work goals [9, 13]. It may however be that these same recommendations would be more credible if delivered by employers, as a means of encouraging and advocating work practice changes required to promote their health and wellbeing.

Our long-term aim was to develop an intervention for delivery by employers as part of an interactive training resource for new employees, and as refresher training for existing employees, who at least occasionally work from home. However, resource constraints and the circumstances of the pandemic led us to assess the content of our intervention as delivered on an individual basis by undergraduate students, with no employer endorsement. We were therefore unable to assess acceptability and feasibility of our intervention content as delivered in the intended setting. The lack of employer endorsement may have been notable in this regard, because organisational buy-in can enhance the acceptability and effectiveness of health-related behavioural interventions among employees [57–59]. For example, employees worry that management will think that if they are standing up more, or sitting less, they will be less productive (see [60]). Organisational endorsement may allay these fears [58]. A homeworking guidance document conceptually similar to ours, launched after we designed our intervention, contains individual-level recommendations while also emphasising the importance of involving managers and supervisors in the delivery of employee health initiatives [61]. The importance of management involvement in the development and delivery of employee behaviour change interventions is increasingly being acknowledged [62].

Limitations of the intervention, and the study more broadly, must be acknowledged. Our aim was to develop an interactive e-learning module, but given time and resource pressures on the project, the clickable-PDF format that we adopted was less interactive than we had hoped. Ideally, future iterations of the intervention would feature greater interactivity, such as quizzes and additional consolidatory learning strategies, to sustain engagement. We investigated elements of acceptability – i.e., adherence and attrition – over a one-week period, but these data are unlikely to capture patterns of engagement and enthusiasm for the intervention over the longer-term.

We opted to assess the acceptability of our intervention only among people who we felt were likely to have sufficient autonomy over their home-working practices to implement our recommendations. We therefore excluded people with caring responsibilities (e.g., parents of young children), on the basis that caring for others while working from home may limit the extent to which participants could modify their work practices as we suggested [63]. However, considering that one in seven workers have caring responsibilities of some sort [64], we recognise the importance of assessing its acceptability among, and potentially further developing our intervention to address the needs of, a more diverse body of office workers.

We drew on theory and evidence to guide intervention content [9, 33], offering recommendations that we felt would motivate home-workers to adopt better work practices and highlight opportunities to incorporate such practices into everyday home-working routines. The strategies that we recommended may however have lacked motivational impact or offered opportunities of little value to participants. Indeed, data were collected ten months after UK stay-home lockdown conditions were imposed (March 2020), so our participants are likely to have had accrued extensive experience of spontaneously learning and adapting their working practices whilst working from home. This may have significantly lessened the impact and acceptability of our intervention content.

Lastly, we delivered the intervention independently of participants’ employers. Although we assumed that our participants had sufficient autonomy to implement our recommendations, the lack of endorsement from employers may have led some participants to question whether the recommended work practice changes were compatible with their work-related goals. It is possible that the same intervention content may have appeared more acceptable when delivered in the intended context (i.e., as an employer-provided staff training module).

## Conclusions

Our intervention aimed to provide guidance on how to work effectively in a way that also shields or improves health behaviours and wellbeing. Participants recognised the need for and value of this information but, likely owing to having spontaneously adapted to working from home during the Covid pandemic, found the information lacked novelty and personal applicability. Adjustments are needed to this intervention to further improve its acceptability prior to progressing to a more rigorous trial. We intend to remove what participants felt was less credible behavioural advice and deliver the remaining guidance elements as part of a broader organisational strategy promoting health when working from home.

## Supplementary Information

**Additional file 1: Additional file 1. **Intervention content: description and component behaviour change techniques (BCTs).

**Additional file 2: Additional file 2. **Intervention content: Screen shot document examples.

**Additional file 3: Additional file 3. **Think-aloud instructions.

**Additional file 4: Additional file 4. **Follow up interview schedule.

## Data Availability

Data used in this study are available from the corresponding author on reasonable request.

## Footnotes

1 ‘Average hours worked’ was not successfully measured and therefore was not included in the analysis.

2 Participants were misled because the PDF viewer for the intervention included all document pages, inclusive of the pop out pages. Therefore displaying a total of 42 pages, when in fact the true page count of the intervention was 15.
