# Supplementary material for: An intervention to promote positive homeworker health and wellbeing through effective home-working practices: a feasibility and acceptability study
Source: BMC Public Health. 2023 Mar 31;23:614. doi: 10.1186/s12889-023-15347-x (PMC10063430; doi:10.1186/s12889-023-15347-x)
Supplement: Supplementary file 1 — Additional file 1: Additional file 1. Intervention content: description and component behaviour change techniques (BCTs). [file 12889_2023_15347_MOESM1_ESM.docx]

**Additional file 1.** Intervention content: description and component behaviour change techniques (BCTs).

| ***Section heading***  ***(pages covered)*** | ***Informational content / behaviour change recommendations ****  ***(PO - pop out information type)*** | ***Specific health behaviour / wellbeing domains targeted*** | ***Intended behaviour change techniques*** |
| --- | --- | --- | --- |
| *‘Getting in the zone’*  *(i.e., managing workspace)*  (3 - 5) | - **Creating a workspace** - highlighting the benefits of having dedicated space in the home for work related tasks (Ahrentzen, 1990). - **Identifying your workspace** - Acknowledging the optional preference of having a single dedicated workspace or using multiple areas of the home for particular tasks + Highlighting the need to build a mental association with spaces for productive work (Keightley et al., 2022)   - **Finding what works for you (PO - *behavioural tip*s)**     - Create a work only space       - E.g. “- *“Try to avoid settings that you associate with leisure activities. Working in bed, for example, can interfere with the lifelong associations you have between the bed and relaxing or sleeping.”*     - Keep away from distraction       - E.g. *“Choose a space where you are unlikely to be distracted – for example, by family or housemates, or by non-work tasks.”*     - Clear the clutter       - E.g. *“If your workspace is in an area you use for non-work tasks – for example, the kitchen table - move all work materials out of sight at the end of your workday.”*     - Make personal space       - E.g. *“create your own personal space and shut out the outside world by, for example, plugging in headphones and listening to music.”*     - Good posture       - E.g. *“Adjust your seat: you should be able to use the keyboard with wrists and forearms straight and level with the floor”* *(***the cited link here has not been removed from the NHS website***)* | Work life balance  Sleep  Posture / DSE | Information about social and environmental consequences; Instruction on how to perform behaviour |
| *‘Striking a balance’*  *(i.e., managing work-life balance)*  *(6 – 10)* | - **Separating work from leisure** – Illustrating the previous benefits of the commute in mentally shifting individuals between work and home as well as promoting physical activity, therefore highlighting the need to alternate ways of shifting between work and leisure time (Jachimowicz et al., 2020).   - **Mental transition (PO – *Quotation – Keightley et al., 2022*)**     - E.g. *“I go for an evening walk – it feels quite good to close your laptop, and kind of ‘leave the office’.”*   - Do something to mark the start and end of your Workday (PO – *Behavioural tip – Keightley et al., 2022)*     - E.g. *Many people find going out for exercise – such as a short walk, run, or cycle – helps to ready them for work.* - **Structuring your day** – Acknowledging working schedules and encouraging ways to segment the working day that promotes physical activity, hydration and reduces sedentary behaviours (Dewitt et al., 2019).   - **Tips for planning your day - (PO – *Behavioural tips – Keightley et al., 2022)***     - Add it to the calendar       - E.g. “- Use your online calendar to reserve chunks of time in your day to focus on specific tasks.”     - Ignore distractions (Kushlev & Dunn, 2015)       - E.g. “Checking and replying to email only at certain times of the day can reduce stress.”     - Reduce unproductive time       - E.g. “*When scheduling meetings, try not to allow too much time. Shorter meetings can be more efficient.”*     - Take breaks       - E.g. *“Schedule regular breaks, either at regular time intervals (eg every hour)”*     - Take a thinking break       - E.g. *“Taking a break and going for a walk, or even just moving around your home, can give you the thinking space you need to take stock, be creative, or solve difficult problems.”* - **Switching off** – Highlight the importance of setting rigid boundaries between work and leisure time through routined time keeping with start and stop times (Sonnentag, 2012).   - **Switching off (PO – *Quotation – Keightley et al., 2022*)**     - E.g. “Alerts pop up all the time. So I just turn all of my notifications off.” - **Getting into sleep mode -** Highlight how mobile phone and late night screen use can interfere with sleep (Quante et al., 2019)   - **Getting into sleep mode (PO – *Research study)***     - E.g. “Simply leaving their devices out of reach each night led them to have better sleep, more energy, and better mood (Mohideen et al., In Press)”. | Work life balance  Physical activity  Sedentary behaviour  Water consumption  Job-satisfaction  Stress  Sleep | Information about emotional consequences; information about health consequences |
| *‘Looking after yourself’*  *(i.e., integrating health promotion into work practices)*   1. – 14) | - **Staying healthy** – Highlight health behaviour risks of a homeworking routine and that healthier behaviours can be integrated into the working day. - **Break up your sitting –** Highlighting the sedentary nature of homeworking (e.g. prolonged sitting & lack of commute) and signalling the need to offset this physical activity loss with at least 2 hours stood a day (Buckley et al., 2015).   - **Tips for breaking up your sitting (PO – *Behavioural tips - Keightley et al., 2022*)**     - Make a stand       - E.g. *“If you can, find a working space in your home that allows you to work while standing”*     - Make a moving habit       - E.g. *“Think about which of your work tasks you can do while standing, or moving around – for example, taking phone calls, or online meetings”*     - Walking meetings       - E.g. *“Video call apps can usually be accessed via a phone, making it possible to attend online meetings while walking.”* - **Eating and drinking –** Indicating the potential for home environments to alter daily eating behaviours, as well as encouraging ways to stay hydrated whilst promoting physical activity (e.g. “Using a small bottle, and refilling it every time it is empty, will require you to walk to the kitchen”   - **Avoid unhealthy snacking (PO – *Behavioural tip - Marteau et al., 2012)***      - Hard to resist       - E.g. *“If you find it hard to resist snacking, try keeping unhealthy snacks in a locked cupboard, or taping the cupboard shut.”* | Sedentary behaviour  Physical activity  Dietary consumption  Diet - Snacking  Water consumption | Information on health consequences |

*Note. PO = Pop out*

* Tip descriptions are not comprehensive. Only text explicitly describing a behaviour change recommendation is included in this table; justifications or explanation of tips are not provided.

*Intended behaviour change techniques – Drawn from the BCT Taxonomy v1 (Michie et al., 2013).*
