# Supplementary material for: An intervention to promote positive homeworker health and wellbeing through effective home-working practices: a feasibility and acceptability study
Source: BMC Public Health. 2023 Mar 31;23:614. doi: 10.1186/s12889-023-15347-x (PMC10063430; doi:10.1186/s12889-023-15347-x)

**Additional file 2.** Intervention content: Screen shot document examples

- Intervention format example – Page 8 – ‘Structuring your day’:


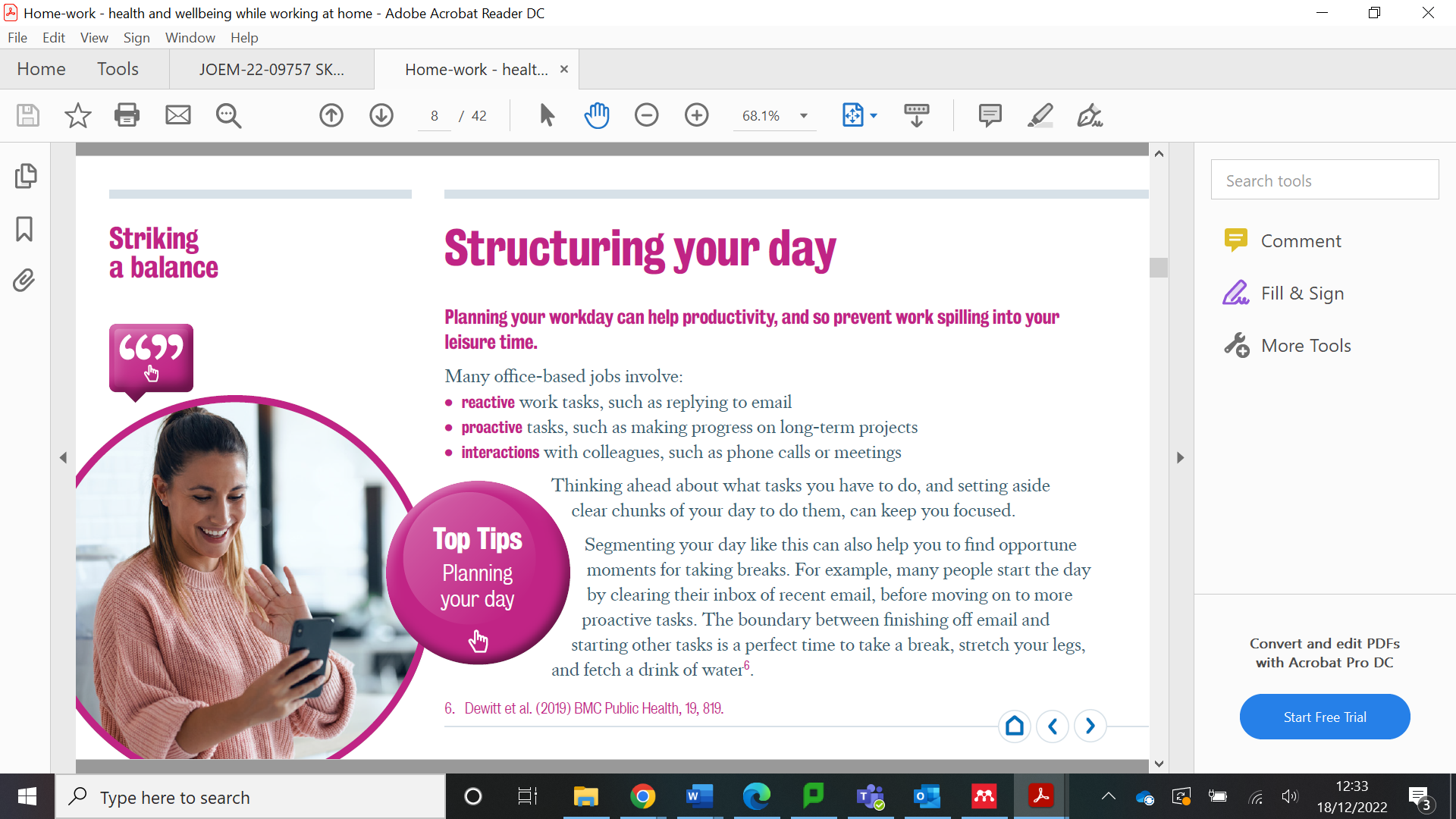


- Page 8 example – Top tips pop out:


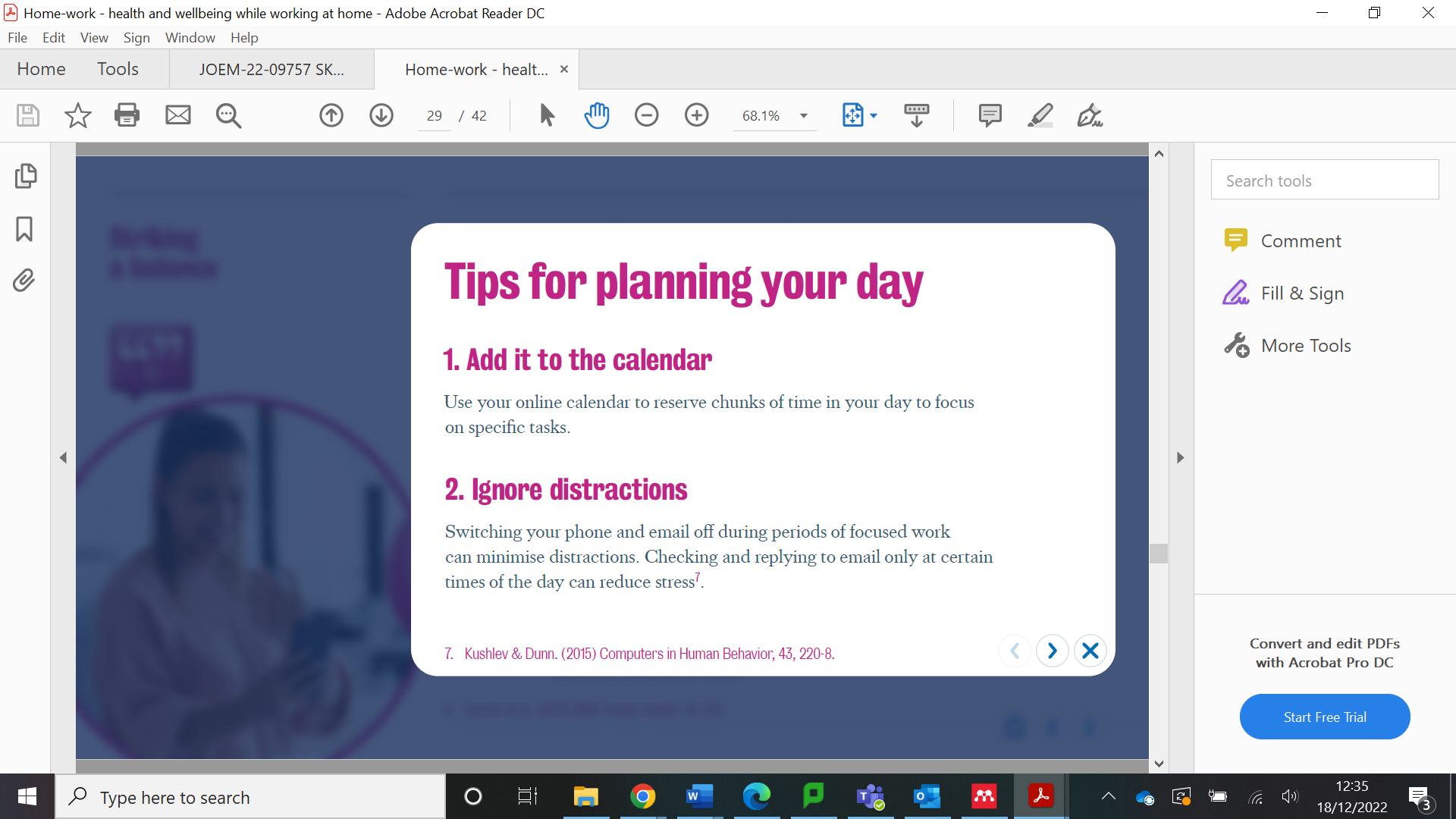


- Page 8 example – Quotation pop out:


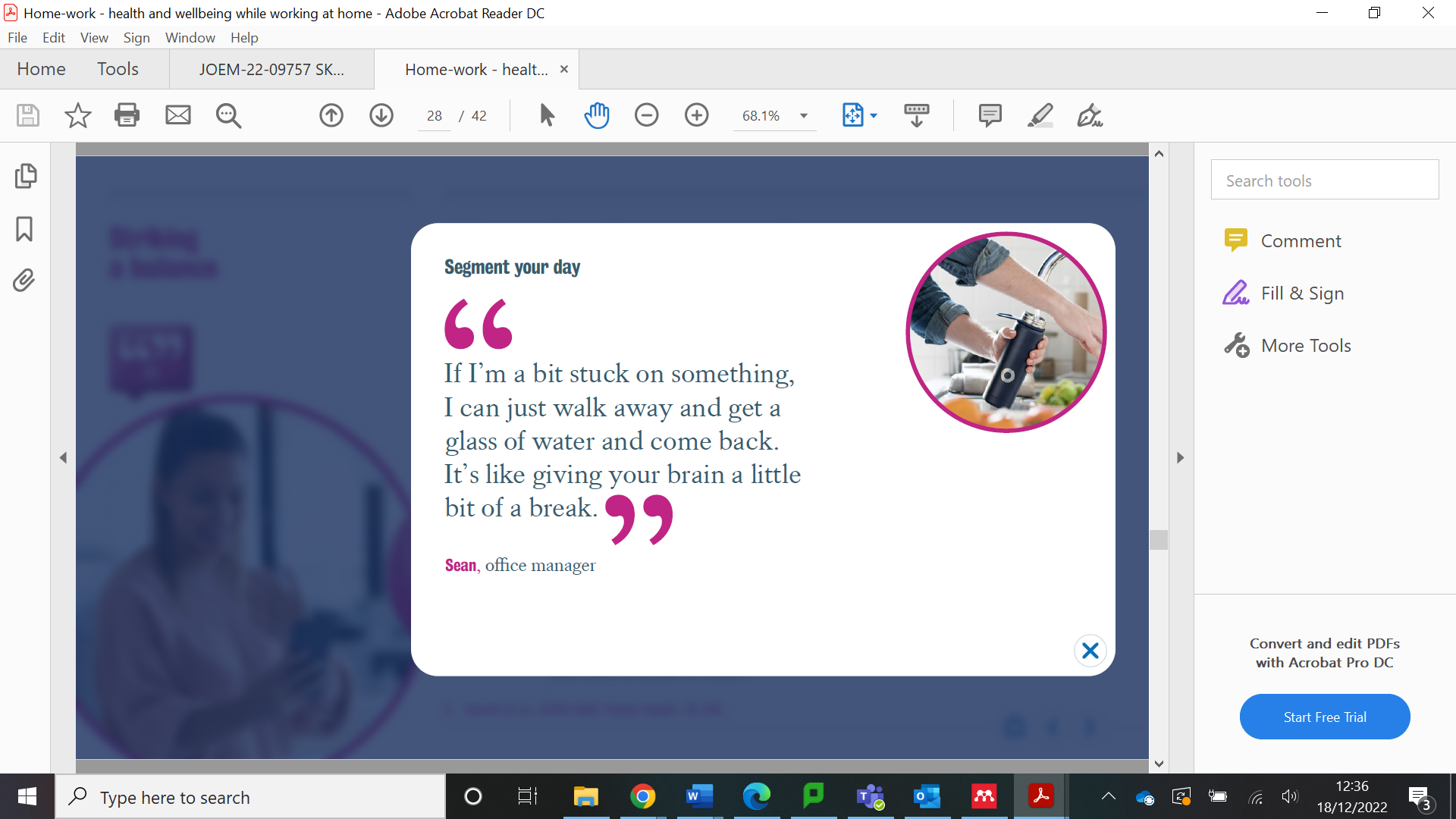

Supplement: Supplementary file 2 — Additional file 2: Additional file 2. Intervention content: Screen shot document examples. [file 12889_2023_15347_MOESM2_ESM.docx]
