# Supplementary material for: An intervention to promote positive homeworker health and wellbeing through effective home-working practices: a feasibility and acceptability study
Source: BMC Public Health. 2023 Mar 31;23:614. doi: 10.1186/s12889-023-15347-x (PMC10063430; doi:10.1186/s12889-023-15347-x)
Supplement: Supplementary file 3 — Additional file 3: Additional file 3. Think-aloud instructions. [file 12889_2023_15347_MOESM3_ESM.docx]

**Additional file 3.** Think-aloud instructions


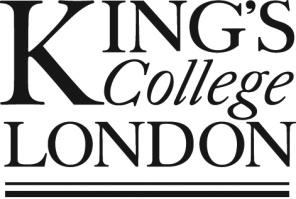


Now we’d like you to read through our information about working from home. As you read each sentence, we would like you to “think aloud”. In other words, we want you to **speak every and any thought that comes to mind as you read through the information, from the beginning to the end of this e-module.** *Don’t plan out what you are saying, or explain what you mean. Instead just freely voice your thoughts as they come to you.*

Throughout the e-module, you will have the opportunity to click on pop up sections, **so please make sure to click on these so you are able to view all the information included** – The icons for these pop ups will look like the ones below:

*
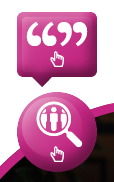

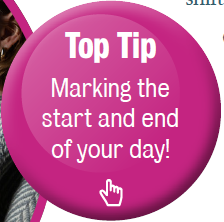
*

Please try to **speak as frequently** and **clearly as possible**. Bear in mind that we need to transcribe this recording and we won’t know which part you are commenting on. *So please read the part you are referring to out loud first and then say what you think about it after.*

Try and be as descriptive as possible as this will allow us to have a better understanding of what you are thinking as you read the sentence. There are no right or wrong answers to this – we are only interested in what you think with regards to the information you’ll be reading.

Next, I am going to turn my camera and my microphone off and will let you talk aloud as you read the information. Please also share your screen so that I can follow which part of the information you are reading. If you stop talking, I will unmute myself just to remind you to carry on talking.

Do you have any questions before we begin?
