# Supplementary material for: An intervention to promote positive homeworker health and wellbeing through effective home-working practices: a feasibility and acceptability study
Source: BMC Public Health. 2023 Mar 31;23:614. doi: 10.1186/s12889-023-15347-x (PMC10063430; doi:10.1186/s12889-023-15347-x)
Supplement: Supplementary file 4 — Additional file 4: Additional file 4. Follow up interview schedule. [file 12889_2023_15347_MOESM4_ESM.docx]

**Additional file 4.** Follow up interview schedule

**2nd interview (one week later)**

Over the past week, how did you get on with the advice that was provided?

PROMPTS:

- Did you make any changes to how you worked?
  - If so, how did you get on with those changes?
  - Do you think the changes that you’ve made will stick, or might you revert back to what you were doing before?
    - IF NO - why do you think you’ll revert back?
  - IF NO - what stood in the way of you making changes?
  - Did you experience any difficulties in following the advice? If so, what?
  - Was there anything we could have done to make it easier for you to follow the advice?

Was there any particular advice that you found particularly useful over this past week?

Did you refer back to the information at any time during the past week?

PROMPTS:

- What information did you refer back to?
- Was it easy or difficult to find?

Knowing what you know now, what advice would you give to someone working from home and would you recommend this module to them? Why/why not?

Are there any additional comments you would like to give us?
